# Effect of pharmacokinetics and pharmacogenomics in adults with allogeneic hematopoietic cell transplantation conditioned with Busulfan

**DOI:** 10.1038/s41409-023-01963-z

**Published:** 2023-04-21

**Authors:** Claire Seydoux, Chakradhara Rao Satyanarayana Uppugunduri, Michael Medinger, Tiago Nava, Joerg Halter, Dominik Heim, Yves Chalandon, Urs Schanz, Gayathri Nair, Nathan Cantoni, Jakob R. Passweg, Marc Ansari

**Affiliations:** 1 Division of Hematology, University Hospital of Basel, Basel, Switzerland and University Basel, Basel, Switzerland; 2 Division of Pediatric Oncology and Hematology, Department of Women, Child and Adolescent, University Geneva Hospitals, Geneva, Switzerland; 3 Cansearch Research Platform for Pediatric Oncology and Hematology, Faculty of Medicine, Department of Pediatrics, Gynecology and Obstetrics, University of Geneva, Geneva, Switzerland; 4 Division of Hematology, Bone Marrow Transplant Unit, University Hospital of Geneva and Faculty of Medicine, University of Geneva, Geneva, Switzerland; 5 Department of Medical Oncology and Hematology, University Hospital of Zurich, Zurich, Switzerland; 6 Division of Oncology, Hematology and Transfusion Medicine, Kantonsspital Aarau, Aarau, Switzerland

**Keywords:** Stem-cell research, Acute myeloid leukaemia, Risk factors, Cancer genomics, Haematopoietic stem cells

## Abstract

Busulfan (Bu) combined with cyclophosphamide (Cy) is commonly used as a myeloablative conditioning regimen for allogeneic hematopoietic cell transplantation (allo-HCT). There is inter-individual variability of Bu pharmacokinetics (PK) and hence in toxicity and efficacy. The introduction of therapeutic drug monitoring (TDM) of Bu has decreased toxicity of the regimen. Hepatic metabolism of Bu is mediated through Glutathione-S-Transferases (GSTs), mainly *GSTA1*. Patients with GSTA1*A variants are considered normal metabolizers and GSTA1*B corresponds to poor metabolism, defined by nucleotide changes at −52 or −69 locus in *GSTA1* promoter region. The aim of the study was to explore the correlation between *GSTA1* polymorphisms and Bu-PK in 60 adult patients receiving an allo-HCT in the BuCyBu clinical study (ClinicalTrials.gov I, ID NCT01779882) comparing the sequence BuCy to CyBu. DNA samples prior to conditioning were genotyped for candidate variants at −52 (rs3957356) and −69 (rs3957357) loci in the *GSTA1 promoter*. Thirty-three % of patients were GSTA1*A*A, 49% GSTA1*A*B and 18% GSTA1*B*B. In GSTA1*A*A patients, median Bu-AUC was 3.6 ± 0.7 mg*h/L, in GSTA1*A*B 4.5 ± 1.6 and in GSTA1*B*B 4.9 ± 1.4 (AUC 35% higher than GSTA1*A*A, *p* = 0.03), with a similar significant correlation with Bu-clearance (*p* = 0.04). The correlation between *GSTA1* polymorphism and AUC remained significant in multivariate linear regression analysis. There was a trend for lower non-relapse mortality (NRM) in patients with low AUC. We could not demonstrate a correlation between *GSTA1* polymorphisms and NRM, acute graft-versus-host disease (aGvHD) in this small cohort, but there is a trend of higher aGvHD incidence in GSTA1*B*B patients.

## Introduction

Busulfan (Bu) and cyclophosphamide (Cy) are commonly used alkylating agents and their efficacy as a myeloablative conditioning regimen for allogeneic hematopoietic cell transplantation (allo-HCT) is well known [1–4]. It has been demonstrated that Bu may affect the hepatic metabolism of Cy, and may therefore increase hepatic toxicity when the combination is given as Busulfan-Cyclophosphamide (BuCy) instead of Cyclophosphamide-Busulfan (CyBu) [5, 6]. A previously published randomized clinical trial (BuCyBu study) suggested that CyBu could be beneficial over BuCy in terms of short-term liver toxicity and long-term outcomes [7]. There is great inter-individual variability of Bu pharmacokinetics (PK) [8–10]. The introduction of Bu therapeutic drug monitoring (TDM) has permitted a reduction in liver toxicity and sinusoidal obstruction syndrome (SOS) incidence. One of the contributing factors to Bu-PK variability, is that hepatic Bu metabolism is mediated by Glutathione-S-Transferases (GSTs) [11–13]. Hypothesis is that some functional polymorphisms of GSTs, specifically the Glutathione-S-Transferase Alpha1 (*GSTA1*) promoter region, may influence enzyme activity and therefore PK and toxicity [14–16]. The two main promoter variants of *GSTA1* consist of GSTA1*A, with individuals showing lower Bu exposure, and GSTA1*B showing higher Bu exposure, but most studies have been done in children [15, 17]. Pharmacogenomics (PG) data may add information to better understand the Bu exposure and thus efficacy or toxicity of Bu individually when associated with PK. The current study aims to investigate the correlation between the two main *GSTA1* promoter polymorphisms (at −52 and −69 loci) and Bu-exposure, as well as the impact of *GSTA1* polymorphisms on clinical outcomes in patients enrolled in the BuCyBu trial (ClinicalTrials.gov I, ID NCT01779882).

### Patients and methods

#### Study cohort and design

This is a translational research project of a prospective multicenter (University Hospitals of Basel and Geneva, Switzerland) randomized trial, comparing the relation between Bu-PK values and *GSTA1* promoter polymorphisms in adult patients receiving BuCy or CyBu as myeloablative conditioning regimen for allo-HCT, 2013 to 2017. DNA was obtained from the Swiss Transplant Cohort and stored in Basel. Analysis of genetic variants and statistical analysis were performed by the CANSEARCH Research Platform in Pediatric Oncology and Hematology of University of Geneva. The study was registered with ClinicalTrials.gov as NCT01779882, approved by Swissmedic (2012DR4164), and the local ethics committee (EKNZ EKBB179/12). The primary endpoint of this study was the correlation between the main *GSTA1* promoter variants and Bu-PK in terms of Bu area-under-the-curve (AUC) and Bu clearance of the first Bu dose. Secondary outcomes were the impact of *GSTA1* on adverse clinical outcomes described in the literature, namely acute GvHD, relapse and NRM at 2 years.

We included adult patients planned for myeloablative conditioning before allo-HCT from an HLA-identical sibling or minimum 10/10 matched unrelated donor who agreed to participate to the study with an informed signed consent. Hematological malignancies were acute myeloid leukemia (AML), chronic myeloid leukemia (CML), and myelodysplastic syndrome (MDS) or myeloproliferative neoplasia (MPN). Exclusion criteria were patients with relevant comorbidities and/or previous abnormal liver function tests within two weeks before first Bu dose. All endpoints were measured from the time of transplantation. Early disease was defined as CR1, intermediate disease was defined as CP1, CR2 or never treated and advanced stage was defined as relapsed or refractory disease, disease persistence, accelerated phase, blast crisis or CP > 1. Acute GvHD was defined as clinically relevent with grade >II. Overall survival was measured as time to death from any cause and NRM was defined as death from any cause without previous relapse or progression. Graft-versus-host-free-relapse-free survival (GRFS) was the earliest occurrence of grade >III of aGvHD, severe cGvHD requiring systemic treatment, relapse or death from any cause after transplant [18]. Of the 70 patients participating in the BuCyBu study [19], 60 had available Bu-PK data and DNA samples and were therefore included in the present study.

The conditioning regimen consisted of either BuCy or CyBu depending on randomization, (i.v. Bu 4x0.8mg/kg for 4 days with a total of 16 doses, followed or preceded by i.v. Cy 60mg/kg for 2 days, see Supplementary Table 1 for detailed treatment*)*. A time interval of 24 h was respected between the infusion of Bu and Cy [6]. Oral UDCA (3x 250 mg daily) and continuous infusion of low-dose heparin (5000 IE/day) was used as SOS prophylaxis and was usually started simultaneously with the conditioning regimen and stopped either after engraftment, when liver values were within normal range or until day+100 for GvHD prophylaxis as per centers’ guidelines. Patients received antiviral prophylaxis with valaciclovir (500mg/day PO) until day+30 (2 years post-HCT in Geneva), prophylaxis against Pneumocystis jirovecii and Toxoplasmosis with trimethoprim/sulfamethoxazole (160/800mg PO, 3 times weekly) at least for 6 months after HCT, and fluconazole (400 mg PO once weekly) as prophylaxis against yeast infections until day+30 (day +100 in Geneva). Most patients did not receive mold-active prophylaxis but were treated empirically or pre-emptively, following a diagnostic-driven approach, based on chest CT scans and serum galactomannan that were regularly performed [20]. GvHD prophylaxis consisted of cyclosporine A (CsA) and methotrexate (MTX) in doses described in Supplementary Table 2, or mycophenolate mofetil (MMF). An addition of anti-T-cell globulin (ATG-Grafalon; Neovii; 35mg/kg total dosis) or alemtuzumab (Campath; Sanofi Genzyme; 20mg for 2 days) was administered if transplant was with an unrelated donor or if donor or recipient were ≥40 years old [21]. Acute GvHD was graded according to the modified Glucksberg criteria [22]. In case of clinically relevant acute GvHD grade ≥II, patients were treated with i.v. corticosteroids (methylprednisolone, 2mg/kg/d)[23].

#### Pharmacokinetic and pharmacogenomic analysis

Bu-AUC was determined with 5 Bu plasma concentrations at different time points (2, 2.5, 3, 4 and 6 hours after the start of the first infusion [24]). Bu dose adjustment according to first AUC (obtained using non-compartmental analysis) was performed from the third or fifth dose onward to achieve a target AUC from 3.65 to 5.48 mg*h/L (i.e 900–1350 μmol/l*min) according to the European Medicines Agency (EMA) therapeutic window. Dosis were adjusted by adding or withholding a 25% dose in patients with AUC higher or lower than 25% of the defined acceptable range. For deviation more than 25%, dosis adjustement was not performed. Centers performing the Bu PK were cross-validated to have comparable analytical estimates.

Regarding PG, the genotyping of six SNPs (rs3957356, rs3957357, rs11964968, rs4715332, rs4715333, rs58912740 [24]) in *GSTA1* promoter region was performed using Sanger sequencing of the entire *GSTA1* promoter region as described previously [17]. PHASE (Version 2.1) was used to resolve the haplotypes including genotype data for six loci of CEU population from 1000 genome project along with the study population [25]. Our population was separated in 3 groups according to global grouping (GSTA1*A*A, GSTA1*A*B and GSTA1*B*B) derived from variant allele presence or absence at −52 (rs3957356) and −69 (rs3957357) loci, irrespective of arm.

#### Statistical analysis

Non-parametric tests (due to non-normal distribution) compared the Bu AUC levels between the groups based on *GSTA1* promoter polymorphisms (Mann-Whitney test or Wilcoxon test). *P* value was adjusted for false discovery rate using Benjamini and Hochberg (B-H) method for number of tests investigated for a specific clinical outcome. Statistical significance was set by a two-sided *p* value < 0.05. Similarly, the demographic characteristics groups were compared between the genotype groups for testing differences in their distribution.

ROC curve analyses was performed to define the cutoff in Bu AUC levels with better sensitivity and specificity to predict NRM at 2 years post HCT, irrespective of treatment arm. Regarding PG, the influence of *GSTA1* on Bu AUC levels was analyzed using a regression model taking into consideration of the following variables (lab values measured within five days before beginning of first Bu dose): albumin, ASAT, ALAT, AP, GGT and Bilirubin levels, *GSTA1* *A*A, *A*B and *B*B, age and weight. The final multivariate model was selected based on the BIC criteria by back elimination. The relation between PG and clinical outcomes was analyzed by cumulative incidence using competing risk model with relapse as a competing risk for non-relapse mortality and death as a competing risk for aGvHD. Clinical outcomes correlation analyses included individuals with no missing data in any of the variables were included in multivariate analyses (*n* = 60). Cumulative incidence was obtained using the cumulative incidence function in the competing risk package (cmprsk) in R [26] and greys test *p* values are provided. Data analyses were carried out using the statistical software R version 3·6.2 with Rcmdr package version 2·6.1 and the survival with cmprsk2 packages.

## Results

### Baseline characteristics

A total of 70 patients were randomized and took part in the initial randomized study and 60 had available DNA and PK samples; of them 30 received CyBu and 30 received BuCy. A total of 36 (60%) patients were male, with a median age of 47.2 years-old at allo-HCT. 45 (75%) patients were treated for AML, 12 (20%) for MDS/MPN and 3 (5%) for CML. This was the second (or more) HCT for 9 patients. At time of transplant, most patients were in early stage (38 patients, 63%), 19 were in intermediate stage and 3 in advanced stage. All but 2 patients received transplant from peripheral blood as stem cell source. Donors were HLA identical siblings in 48.3 % of the cases and 10/10 matched unrelated in 51.7%. GvHD prophylaxis consisted of CsA in 56/60, MTX in 57/60 and MMF in 2/60 patients. ATG or T-cell depletion was given in a total of 42 (70%) patients. A total of 21 (35%) patients were GSTA1*A*A, 28 (47%) were GSTA1*A*B and 11 (18%) were GSTA1*B*B. Distribution of all patients’ characteristics according to PG groups is displayed in Table 1.

**Table 1:** Patient, disease and transplant characteristics according to PG.

Patient’s characteristics	GSTA1*A*A (*n* = 21)	GSTA1*A*B (*n* = 28)	GSTA1*B*B (*n* = 11)
Age (median, years; range)	48.0 (25.4–65.1)	45.4 (20.7–64.6)	52.9 (32.6–62.0)
Gender male (n, %)	13 (61.9)	16 (57.1)	7 (63.6)
Weight (median, kg; range)	68.4 (50.6–96.9)	74.5 (52.9–106.1)	79.7 (54.2–100.5)
Disease			
AML (n, %)	17 (81.0)	23 (82.1)	5 (45.5)
MDS/MPN (n, %)	3 (14.3)	5 (17.9)	4 (36.4)
CML (n, %)	1 (4.7)	0	2 (18.2)
Disease status			
Early disease (n, %)	15 (71.4)	16 (57.2)	7 (63.6)
Intermediate disease (n, %)	6 (28.6)	9 (32.1)	4 (36.4)
Advanced disease (n, %)	0	3 (10.7)	0
Donor characteristics			
Donor age (median, years; range)^a^	28.4 (19.7–58.0)	41.7 (24.1–58.9)	44.0 (24.1–58.9)
Donor female/ recipient male (n, %)	3 (14.2)	6 (21.4)	4 (36.3)
HLA-identical sibling (n, %)	8 (38.1)	16 (57.1)	5 (45.5)
HLA matched unrelated (n, %)	13 (61.9)	12 (42.9)	6 (54.5)
Stem cell source peripheral blood (n, %)	21 (100)	27 (96.4)	10 (96.4)
CMV status			
Donor neg / patient pos (n, %)	7 (33.3)	13 (46.4)	4 (36.4)
Treatment arm			
BuCy (n, %)	10 (47.6)	15 (53.6)	5 (45.5)
CyBu (n, %)	11 (52.4)	13 (46.4)	6 (54.5)
GvHD prophylaxis			
ATG or t-cell depletion (n, %)	19 (90.5)	14 (50)	9 (81.8)
MTX (n, %)	18 (85.7)	28 (100)	11 (100)
CSA (n, %)	17 (81.0)	28 (100)	11 (100)
MMF (n, %)	0	1 (3.6)	1 (9.1)
KPS score			
90–100 % (n, %)	19 (90.5)	24 (76.7)	10 (90.9)
<80% (n, %)	2 (9.5)	4 (14.3)	1 (9.1)
Pharmacokinetics			
Bu AUC 1st dose (median; mg*h/l, SD)	3.6 (0.8)	4.3 (1.6)	4.9 (1.3)
Bu AUC < 3.65 mg*h/L (n, %)	13 (62)	8 (29)	4 (36)
Bu AUC 3.65–5.48 mg*h/L (n, %)	8 (38)	12 (43)	5 (46)
Bu AUC > 5.48 mg*h/L (n, %)	0	8 (29)	2 (18)

*allo-HCT* allogeneic cell transplantation, *CMV* cytomegalovirus, *AML* acute myeloid leukemia, *MDS* myelodysplastic syndrome, *MPN* myeloproliferative neoplasm, *CML* chronic lymphocytic leukemia, *KPS* Karnovsky Performance Score, *GvHD* graft-versus-host-disease, *ATG* anti-thymocyte globulin, *CyA* cyclosporine A, *MTX* methotrexate, *MMF* mycophenolate mofetil, *SD* standard deviation.

^a^Three missing values.

### Pharmacokinetics

**Fig. 1:**
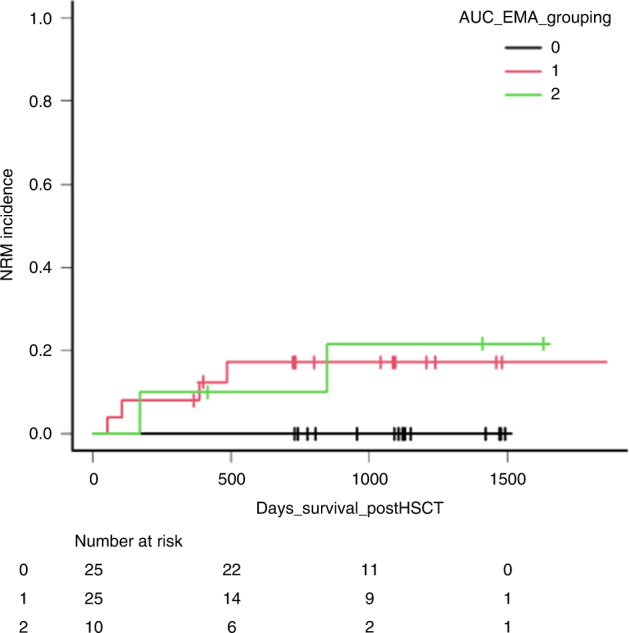
Non-relapse mortality according to Bu-AUC. 0 = low-AUC; 1 = target AUC; 2 = high-AUC.

Median AUC was 4.45 ± 1.4 mg*h/L in our population, there was no significant difference according to treatment arm, but CyBu tends to show lower AUC value (median 4.57 ± 1.12 mg*h/L) in BuCy versus 4.34 ± 1.63 mg*h/L in CyBu (Supplementary Fig. 1*)*. Median clearance was 3.21ml/min/kg. Patients in lower AUC (i.e AUC < 3.65 mg*h/L) showed a trend of lower NRM, with a cumulative incidence of 0%, as compared to 17.2% (95% CI: 5–35.3%) in target (AUC from 3.65 to 5.48 mg*h/L) and 10% (5–37.4%) in high AUC (>5.48 mg*h/L) (*p* = 0.08, Fig. 1). In ROC analysis for NRM time to event analyses indicated an AUC of 4.34 mg*h/L had a 62% specificity and 100% sensitivity for NRM (*p* = 0.001).

### Pharmacogenomics

**Fig. 2:**
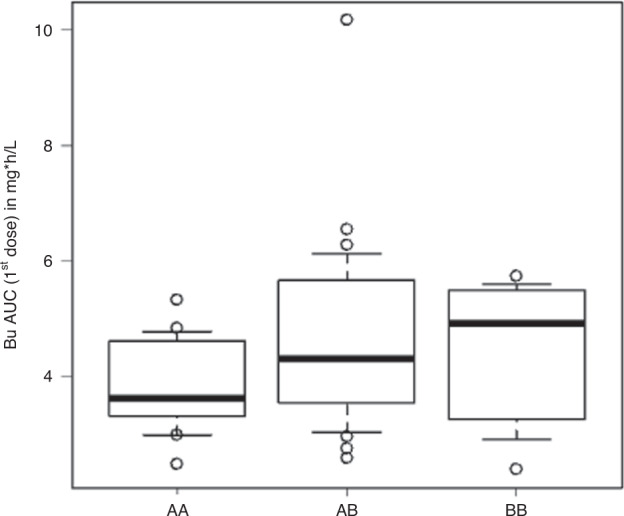
Correlation between Bu-AUC in mg*h/L and GSTA1 genetic variants A*A, A*B and B*B.

**Fig. 3:**
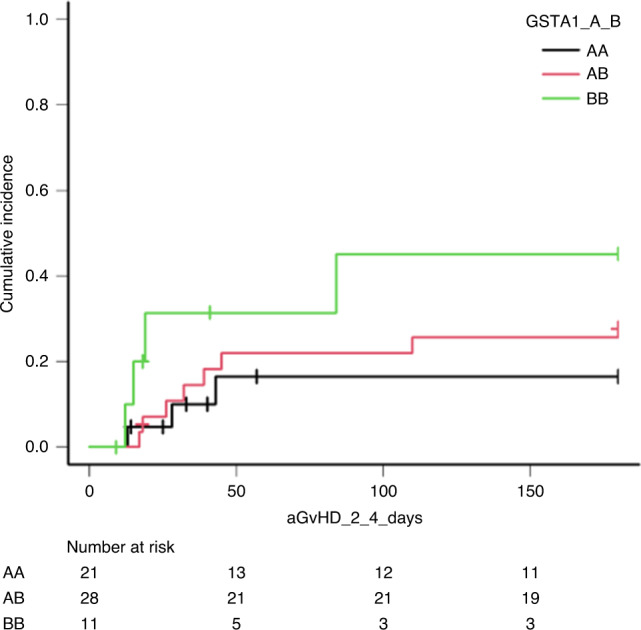
Correlation between aGvHD incidence and GSTA1 genetic variants A*A, A*B and B*B.

GSTA1*A*A patients had a median Bu-AUC of 3.6 mg*h/L, GSTA1*A*B of 4.3 and GSTA1*B*B of 4.9 (Table 1*;* Fig. 2*;* *p* = 0.03). Median ± SD clearance was 3.6 ± 1.3 ml/min/kg, 2.7 ± 1.6 ml/min/kg and 2.7 ± 1.1 ml/min/kg in GSTA1*A*A, GSTA1*A*B and GSTA1*B*B, respectively (*p* = 0.04). After multivariate linear regression, carrying a GSTA1*B allele (either GSTA1*A*B or *B*B) remained a positive predictor for AUC, associated with an AUC reduction of 20% (*p* = 0.02). There was a higher aGvHD grade >2 incidence in GSTA1*B*B (45%: 13–73.3%) as compared to GSTA1*A*A (16.4%: 4–36.5%) or GSTA1*A*B (29.4%: 13.9–46.8%) with a HR of 1.6 (0.6–2.4, *p* = 0.2), shown in Fig. 3. There were no significant differences in overall survival, NRM, GRFS and relapse by *GSTA1* polymorphisms (*p* values: 0.4, 0.7, 0.8 and 0.7; Supplementary Figs. 2, 3, 4 and 5).

## Discussion

This is a translational project of a randomized clinical trial, comparing the impact of the order of application of busulfan and cyclophosphamide used as conditioning regimen for allo-HSCT in adult patients, suggesting a small clinical benefit of CyBu over BuCy. In this project analyzing pharmacogenomics and pharmacokinetics, we show an association between *GSTA1* variants and PK, with low AUC in GSTA1*A*A, and high AUC in GSTA1*A*B and GSTA1*B*B, as well as a trend of higher aGvHD incidence in GSTA1*B*B variants, irrespective of treatment arm.

Incidence of organ toxicity, specifically SOS, has been reduced with the introduction of TDM in the early 2000’s, but still remains at 2–5% [8, 27]. In patients with low Bu metabolism, organ toxicity may be induced even after the first dose of Bu and further dose adjustment may not be efficient, as the subsequent Bu-AUC values remain high [15, 28]. This has led to search for predictors for Bu-PK and therefore NRM. Most studies investigating the relation between Bu-PK and PG are from pediatric populations, only some have been done in adults [14, 29, 30].

Epidemiology of *GSTA1* variants varies in different ethnicities. European GSTA1*A prevalence is about 60% and GSTA1*B 40%, depending on studies [17]. Lately, additional SNPs (other than −52 and −69) have been discovered, influencing the promoter of *GSTA1* and hence enzyme function. *Ansari and al*. have used four additional SNPs (rs11964968, rs4715332, rs4715333, rs58912740 in *GSTA1 promoter)* in a multicenter pediatric population, where some normal metabolizers were re-classified into intermediate metabolizers or ultra-rapid metabolizers creating refined *GSTA1* metabolic groups [15]. Loci −631 and −1142 (rs4715333 and rs58912740) showed highest enzyme activity among GSTA1*A and loci −513 (rs11964968) showed the lowest enzyme activity among GSTA1*B. This refined and more detailed grouping might be interesting for further exploration in adults with a larger patient group (data with four additional SNPs not shown). Our Bu-PK values were slightly higher than described in a meta-analysis by *Kim et al*., where median AUC in GSTA1*A*B* and B*B pooled individuals was 999 µmol/l*min (4.10 mg*h/L) versus 956 µmol/l*min (3.92 mg*h/L) in GSTA1*A*A individuals, though this meta-analysis comprised children and adults with different conditioning regimens [13]. In our study, one allele GSTA1*B could actually be sufficient to increase AUC by 16%, which may be clinically meaningful in patients with AUC at the extremes of the therapeutic window.

Regarding clinical outcome and *GST* polymorphism, most studies are again done in pediatric populations and results are controversial. Most of them show better long-term outcome in GSTA1*A*A carriers, with better event-free survival and lower mortality without impact on the relapse rate [15, 28, 29]. Better outcome in rapid metabolizers (GSTA1*A*A) is described even if they have a Bu-AUC below the target range [15]. Rapid metabolizing might therefore be an overall protective factor independently of low PK, but our population was too small to show such a correlation at long-term. In fact, PG might play a role in NRM, but the most important predictor factor still remains PK. Better outcome in low-AUC as shown here has also been found in a recent retrospective study, in which lower NRM was seen in patients with AUC < 900 µmol/l*min (i.e 3.65 mg*h/L) and a theoretical ideal cut-off range of 962 µmol/l*min [31]. This questions the rationale for dose increase when patients are below the AUC target range.

Results concerning the association between *GSTA1* polymorphisms and aGvHD are more questionable, as it was proposed earlier that GSTA1*A*A may be an independent protective factor against aGvHD [15, 30], but these results were not replicated in other studies [28, 32]. *GSTA1* may have direct impact on the cell protection as demonstrated in earlier reports where GSTA1*B*B individuals were at higher risk of developing treatment related toxicities even within the AUC target window [15, 21]. Actually, PG and PK may inform in a complementary way, with an overall higher toxicity in poor metabolizers and with influence on aGvHD incidence. Other genetic variants of GST, such as Glutathione-S-Transferase Mu1 (GSTM1), Pi1 (GSTP1) and Theta (GSTT1) also participate in the conjugation of Bu with GST [13, 22, 23], e.g. *GSTM1* absence of protein due to gene deletion have a stronger association with relapse than PK [13, 20, 22].

Last regarding Cyclophosphamide, Ekhart et al. did not find an association between *GSTA1* and the metabolism of the drug, though the conditioning regimen given comprised Cy, thiotepa and carboplatin [33]. Although Cy metabolism involves GSTs especially in eliminating active Cy metabolites, the accumulation of Cy toxic metabolites could be only triggered when associated with Bu [28] increased in GSTA1*B*B diplotype carriers. A 24h hour interval between the 2 drugs is therefore recommended to limit NRM [6]. An association between *GSTA1* and Cy-PK was also described in patients with lupus nephritis, with poorer response rate in GSTA1*A*A [34]. Our study was limited by the initial sample size set by the RCT and derived by a previous retrospective study [35]. Another limit is the heterogeneity of our population, with different hematological neoplasms, disease stages, order of application and aGvHD prophylaxis. Larger clinical studies are warranted.

## Conclusions

In conclusion, we demonstrate a positive association between pharmacokinetics and pharmacogenomics, with higher AUC and lower clearance in GSTA1*B*B as compared to GSTA1*A*A. Regarding clinical outcomes, we see a trend of higher aGvHD incidence in GSTA1*B*B patients. These genetic variants, among others, could be future predictive factors of outcome in patients with allo-HCT, but larger studies are needed. This suggests that PG added to TDM may optimize Bu safety and efficacy profile when used in intensive chemotherapy regimens.

### Supplementary Information

Supplementary Figure

## Data Availability

The datasets generated during and/or analysed during the current study are available from the corresponding author on reasonable request.
