# Correction: Effect of pharmacokinetics and pharmacogenomics in adults with allogeneic hematopoietic cell transplantation conditioned with Busulfan

**DOI:** 10.1038/s41409-023-02166-2

**Published:** 2023-12-21

**Authors:** Claire Seydoux, Chakradhara Rao Satyanarayana Uppugunduri, Michael Medinger, Tiago Nava, Joerg Halter, Dominik Heim, Yves Chalandon, Urs Schanz, Gayathri Nair, Nathan Cantoni, Jakob R. Passweg, Marc Ansari

**Affiliations:** 1 Division of Hematology, University Hospital of Basel, Basel, Switzerland and University Basel, Basel, Switzerland; 2 Division of Pediatric Oncology and Hematology, Department of Women, Child and Adolescent, University Geneva Hospitals, Geneva, Switzerland; 3 Cansearch Research Platform for Pediatric Oncology and Hematology, Faculty of Medicine, Department of Pediatrics, Gynecology and Obstetrics, University of Geneva, Geneva, Switzerland; 4 Division of Hematology, Bone Marrow Transplant Unit, University Hospital of Geneva and Faculty of Medicine, University of Geneva, Geneva, Switzerland; 5 Department of Medical Oncology and Hematology, University Hospital of Zurich, Zurich, Switzerland; 6 Division of Oncology, Hematology and Transfusion Medicine, Kantonsspital Aarau, Aarau, Switzerland

**Keywords:** Stem-cell research, Acute myeloid leukaemia, Risk factors, Cancer genomics, Haematopoietic stem cells

Correction to: *Bone Marrow Transplantation* 10.1038/s41409-023-01963-z, published online 21 April 2023

The authors regret to inform that there were errors on page 813 and 814 of the published article.

Error 1: p. 813, line 7 The sentence “(…) a target AUC from 3.65 to 5.48 mg*h/L (i.e., 900–1350 µmol/l*min) according to the European Medicines Agency (EMA) therapeutic window” should be corrected to “(…) a target AUC from 3.65 to 5.48 mg*h/L (i.e., 900–1350 µmol/l*min).” Explanation: the EMA therapeutic window is from 900 to 1500 µmol/l*min

Error 1: p. 813, line 7 The sentence “(…) a target AUC from 3.65 to 5.48 mg*h/L (i.e., 900–1350 µmol/l*min) according to the European Medicines Agency (EMA) therapeutic window” should be corrected to “(…) a target AUC from 3.65 to 5.48 mg*h/L (i.e., 900–1350 µmol/l*min).” Explanation: the EMA therapeutic window is from 900 to 1500 µmol/l*min

Error 3: pg 814, Figure 1 On Figure 1. legend “AUC_EMA_grouping” should be corrected to “AUC_Windows”, and “0 = low-AUC, 1 = target-AUC, 2 = high-AUC” Should be read as 0 = AUC below 3.65 mg*h/L, 1 = AUC between 3.65 and 5.48 mg*h/L, 2 = AUC above 5.48 mg*h/L”

The authors take full responsibility for this oversight and sincerely apologize for any confusion it may have caused to the scientific community.

