# Supplementary material for: Effect of pharmacokinetics and pharmacogenomics in adults with allogeneic hematopoietic cell transplantation conditioned with Busulfan
Source: Bone Marrow Transplant. 2023 Apr 21;58(7):811–6. doi: 10.1038/s41409-023-01963-z (PMC10325946; doi:10.1038/s41409-023-01963-z)
Supplement: Supplementary file 1 — Supplementary Figure [file 41409_2023_1963_MOESM1_ESM.pdf]

Supplementary Figure 1. Bu-AUC according to treatment arm

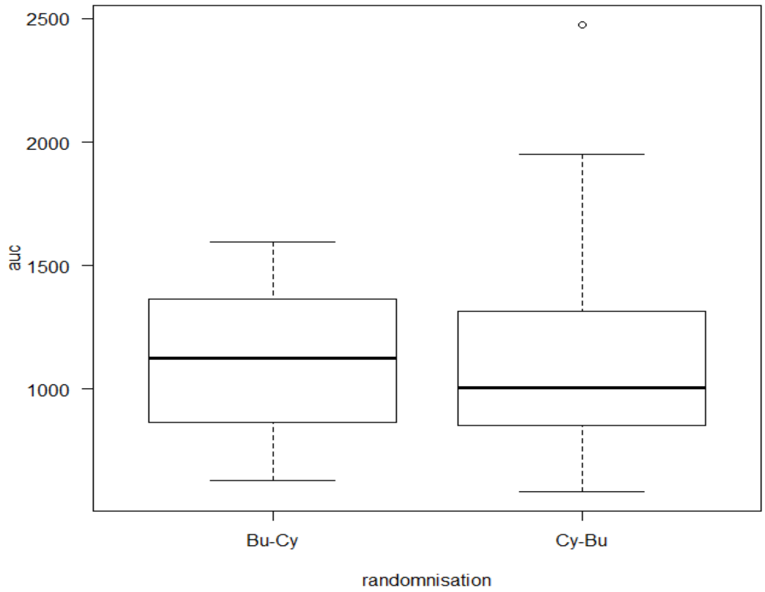

Supplementary Figure 2. Correlation between overall survival incidence and GSTA1 genetic variants
*A*A –*
*A*B - -*
*B*B ···*

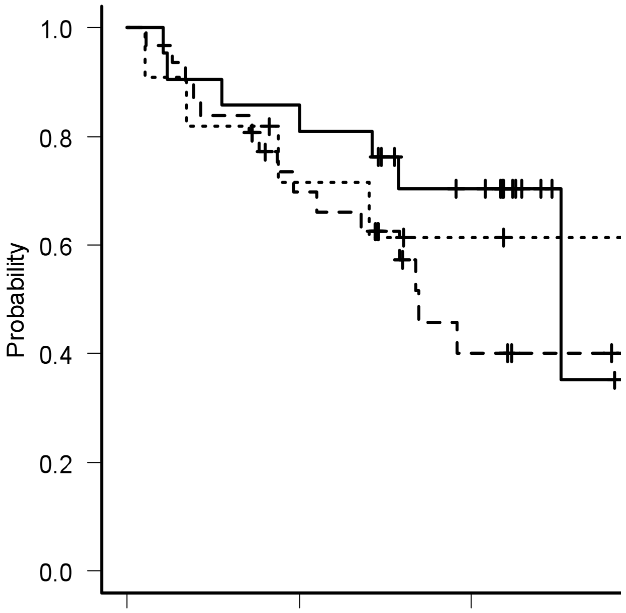

Supplementary Figure 3. Correlation between NRM incidence and GSTA1 genetic variants
*A*A –*
*A*B - -*
*B*B ···*

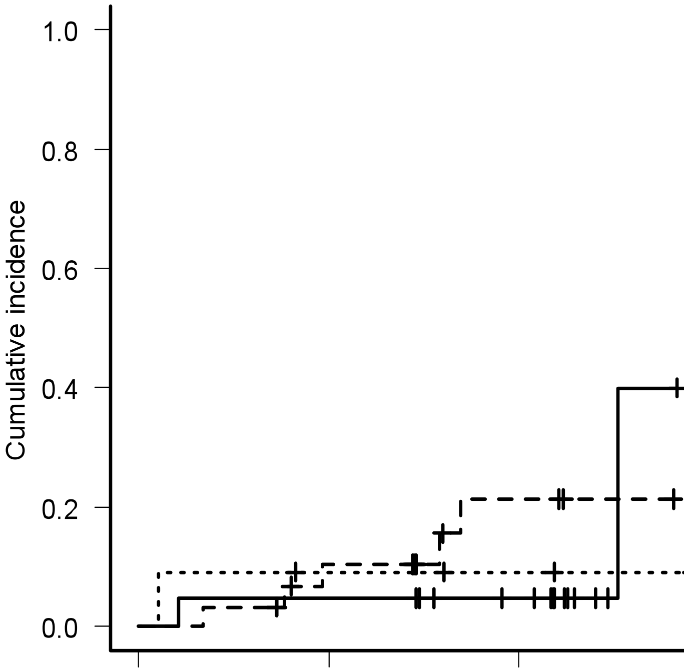

Supplementary Figure 4. Correlation between GRFS incidence and GSTA1 genetic variants
*A*A –*
*A*B - -*
*B*B ···*

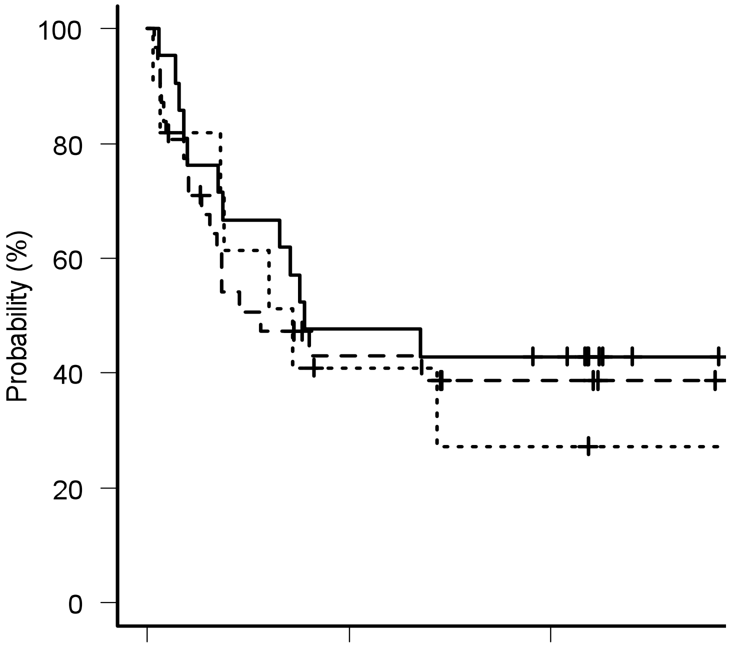

Supplementary Figure 5. Correlation between relapse incidence and GSTA1 genetic variants
*A*A –*
*A*B - -*
*B*B ···*

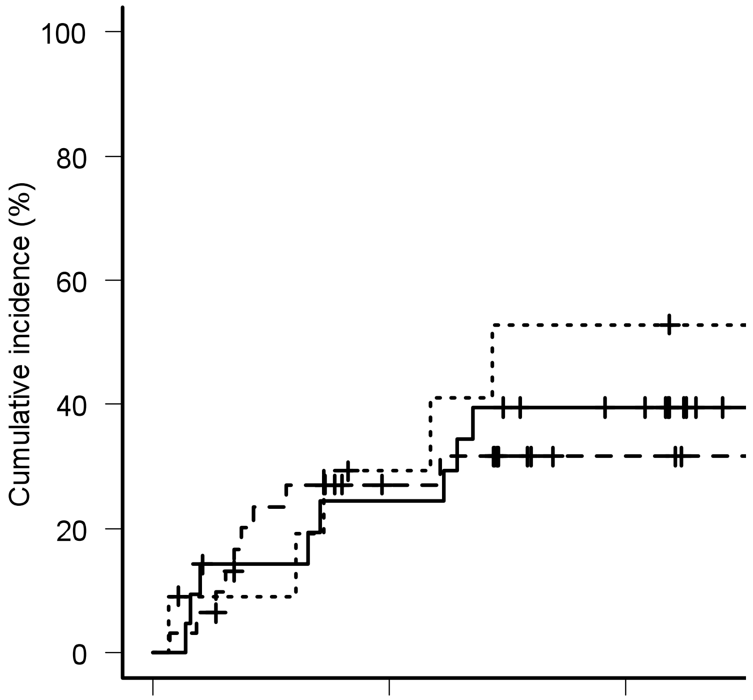

Supplementary Table 1 : Chemotherapeutic protocol according to treatment arm

Chemotherapy	Day -7	Day -6	Day -5	Day -4	Day -3	Day -2	Day -1	Day 0
**BuCy**	i.v Bu 4x0.8mg/kg in NaCl 0.9% over 2h (total 16 doses)[1]				i.v Cy 60mg/kg in 5% G5 over 1h			Allo-HCT
**CyBu**	i.v Cy 60mg/kg in 5% G5 over 1h		i.v Bu 4x0.8mg/kg in NaCl 0.9% over 2h (total 16 doses)[1]					Allo-HCT

[1] First infusion started on the evening of day -8 so that the 24h interval time between Bu and Cy was respected (6).

Supplementary Table 2 : GvHD prophylaxis

GvHD prophylaxis	Day -3	Day -2	Day -1	Day 0	Day +1	Day +2	Day +3	Day +6
**Methotrexate**					i.v 15mg/m^2		i.v 10mg/m^2	i.v 10mg/m^2
**Cyclosporine A**[1]	i.v CsA 2x3mg/kg/day							

[1] Adjusted after first dose to target blood levels of 150-200 μg/L, tapered over 6 months
